# A novel 4.25 kb heterozygous deletion in *PAX6* in a Chinese Han family with congenital aniridia combined with cataract and nystagmus

**DOI:** 10.1186/s12886-021-02120-0

**Published:** 2021-10-05

**Authors:** Tianwei Qian, Chong Chen, Caihua Li, Qiaoyun Gong, Kun Liu, Gao Wang, Isabelle Schrauwen, Xun Xu

**Affiliations:** 1 Department of Ophthalmology, Shanghai General Hospital, Shanghai Jiao Tong University, No. 100 Haining Rd., Shanghai, 200080 China; 2 National Clinical Research Center for Eye Diseases, Shanghai, China; 3 Shanghai Key Laboratory of Ocular Fundus Diseases, Shanghai, China; 4 Shanghai Engineering Center for Visual Science and Photomedicine, Shanghai, China; 5 Shanghai Engineering Center for Precise Diagnosis and Treatment of Eye Disease, Shanghai, China; 6 Department of Neurology, Columbia University Medical Center, 630W 168th St, New York, NY 10032 USA; 7 Singapore Eye Research Institute, Singapore National Eye Centre, Singapore, Singapore; 8 Genesky Biotechnologies Inc, Shanghai, China

**Keywords:** Congenital aniridia, PAX6, Deletion, Copy number variant

## Abstract

**Background:**

The aim of this study is to identify the genetic defect in a Chinese family with congenital aniridia combined with cataract and nystagmus.

**Methods:**

Complete ophthalmic examinations, including slit-lamp biomicroscopy, dilated indirect ophthalmoscopy, anterior segment photography, and anterior segment optical coherence tomography (OCT) were performed. Blood samples were collected from all family members and genomic DNA was extracted. Genome sequencing was performed in all family members and Sanger sequencing was used to verify variant breakpoints.

**Results:**

All the thirteen members in this Chinese family, including seven patients and six normal people, were recruited in this study. The ophthalmic examination of affected patients in this family was consistent with congenital aniridia combined with cataract and nystagmus. A novel heterozygous deletion (NC_000011.10:g.31802307_31806556del) containing the 5′ region of *PAX6* gene was detected that segregated with the disease.

**Conclusion:**

We detected a novel deletion in *PAX6* responsible for congenital aniridia in the affected individuals of this Chinese family. The novel 4.25 kb deletion in *PAX6* gene of our study would further broaden the genetic defects of *PAX6* associated with congenital aniridia.

**Supplementary Information:**

The online version contains supplementary material available at 10.1186/s12886-021-02120-0.

## Background

Aniridia (Online Mendelian Inheritance in Man identifier, OMIM, 106210) is a rare, congenital ocular disorder with the characteristics of partial or complete absence of the iris. Aniridia occurs in approximately 1/64,000 to 1/96,000 live births and is primarily characterized by iris hypoplasia [1, 2]. Two-thirds of aniridia cases have an obvious hereditary history with autosomal dominant inheritance, complete penetrance and variable expressivity, while the remaining cases refer to sporadic cases [3–5]. Aniridia can occur isolated, as part of WAGR (Wilms tumor, aniridia, genitourinary disorders, and retardation) syndrome, WAGRO syndrome (WAGR and obesity), or other associated syndromes [6, 7]. In addition to the variable iris hypoplasia, congenital aniridia is usually accompanied with lens opacity or dislocation, nystagmus, glaucoma, aniridia-related keratopathy.

Paired box gene-6 (*PAX6*, OMIM: 607108), a member of the paired box gene family located on chromosome 11p13, was identified as a candidate gene for aniridia, spanning about 22 kb and encoding a transcription factor that contains two conserved DNA binding domains (a paired box and a paired type homeobox) [8–11]. This gene plays an essential role in eye development, as well as brain, spinal cord and pancreas [12]. Most congenital aniridia cases are caused by variants in *PAX6* [13–15]. Prior to the current study, according to the Human PAX6 Allelic Variant Database (LOVD PAX6 database, version 180,804) (http://lsdb.hgu.mrc.ac.uk/home.php?select_db=PAX6), 491 unique variants of *PAX6* have been identified. Most of these variants are frameshift variants, splice site variants, or nonsense variants, which have been considered to produce truncated proteins or result in loss-of-function due to nonsense mediated decay, while other variants were missense [16, 17].

In this study, we performed genome sequencing to identify the molecular cause of congenital aniridia in a Han Chinese family to further investigate the genetic and phenotypic spectrum of congenital aniridias.

## Methods

### Subject recruitment and clinical examination

**Fig. 1:**
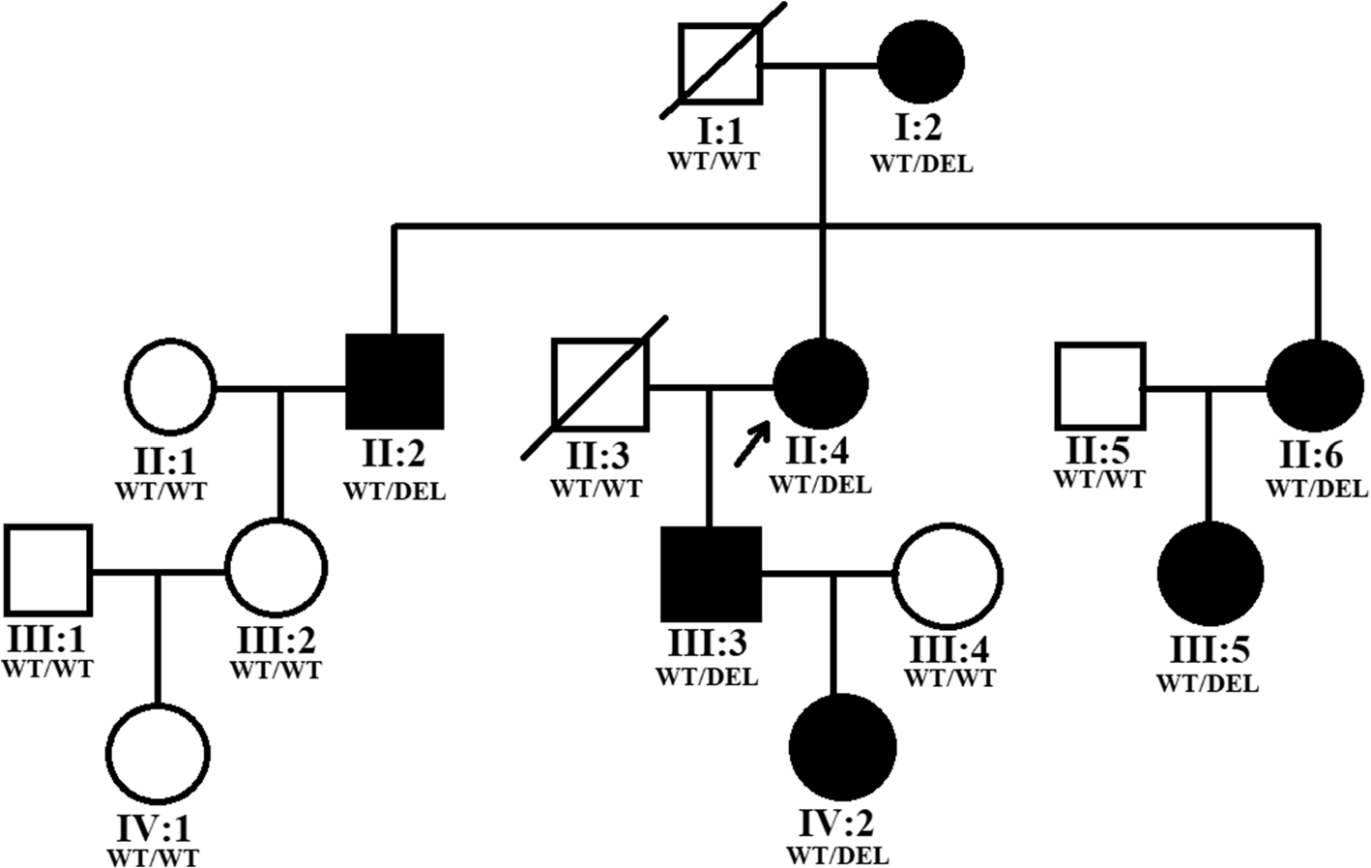
Pedigree of the four-generation family with congenital aniridia. Solid symbols indicate affected individuals, and open symbols indicate unaffected individuals. Arrow indicates the proband of this family

A four-generation family with aniridia was recruited in the Shanghai General Hospital in Shanghai, China. Thirteen family members of this family (Fig. 1) took part in this study. This study was conducted in accordance with the Declaration of Helsinki and was approved by the ethics committee of Shanghai General Hospital. Informed consent was obtained from each participant. Seven of the 13 family members were diagnosed with congenital aniridia. No consanguinity was present in the family. Each family member received complete and comprehensive clinical and ophthalmic examination, including visual acuity test, intraocular pressure (IOP) measurement, anterior segment examination, slit lamp examination, fundus exam and orthoptic evaluation, as well as the examination of physical malformations and neurological deficits. In addition, 300 ethnically matched healthy individuals with no direct or collateral ties and no related phenotypes and systemic underlying diseases were recruited.

### DNA preparation

Genomic DNA was extracted from peripheral blood using the TruSeq DNA LT Sample Prep kit (Illumina, San Diego, CA) according to the manufacturer’s protocol. DNA samples were stored at − 20 °C until used, and DNA integrity was evaluated by 1% agarose gel electrophoresis.

### Whole-genome sequencing

Whole-genome sequencing (WGS) was performed in all 13 family members. The libraries were constructed with TruSeq Nano DNA LT Sample Prepararion Kit (Illumina, San Diego, CA, USA). Briefly, the genomic DNA was sheared into fragments with length ~ 350 bp using S220 Focused-ultrasonicators (Covaris, USA). Adapters were ligated onto the 3′ end of the sheared fragments. After polymerase chain reaction (PCR) amplification and purification, the final libraries were sequenced on the Illumina sequencing platform HiSeq X Ten platform (Illumina Inc., San Diego, CA, USA) and 150 bp paired-end reads were generated. The average sequencing depth was at least 30 × .

### Routine whole-genome sequencing analysis

The raw reads were subjected to a quality check and then filtered by fastp (https://github.com/OpenGene/fastp). Reads were aligned to hg38 using SpeedSeq [18]. Single nucleotide variants and insertions/deletions (indels) calling were performed using Genome Analysis Toolkit v2.1 [19]. Structural variants and copy number variants were analyzed in SpeedSeq [18]. Annotations of single nucleotide variants, indels, structural variants and copy number variants were performed with ANNOVAR [20]. Variant filtering was performed as illustrated in Supplementary 1.

### Real time-polymerase chain reaction

RT-PCR was accomplished using the FastStart Universal SYBR Green Master (Rox) (Roche) in the ABI PRISM® 7300 real time-PCR system (Applied Biosystem, Foster City, CA, USA). POLR2A, RPP14 and TBX15 were used as endogenous controls. We used melting curves to monitor non-specific amplifications. Relative expression level was computed using 2-ΔΔCt method. The primer sequences used were 5′-TCCACGGGGCTCGAATATGG-3′ (forward) and 5′-ACCTCGGTTGGGAGTTCAGG-3 (reverse) for Exon 3, and 5′-AATCTTCTGCCGGGTGGAGT-3′ (forward) and 5′- TTTCCTCAGGTCACAGCGGA-3 (reverse) for Exon 4, separately.

### Variant validation

In order to identify the exact breakpoints of the deletion in *PAX6* gene after WGS analysis, primers were designed in the region surrounding the deletion using Primer3 software (version 4.0, http://bioinfo.ut.ee/primer3-0.4.0/). PCR primer pairs and amplification conditions are available upon request. PCR products were checked by 1% agarose gel electrophoresis and purified with SAP-Exon I kit (USB, USA). Purified PCR products were directly sequenced in both forward and reverse directions using an ABI 3730xl genetic analyzer (Applied Biosystems, Foster City, CA, USA) per manufacturer’s instructions. DNA sequences were analyzed using Chromas (version 2.22) and DNAMAN (version 7) software. The primer sequences were 5′-TAAATTTATTTTTGTGCTGACCTTG-3′ (forward) and 5′- ATTTCAGGCAAGTTCTGTGGTG − 3 (reverse) for the *PAX6* gene.

## Results

### Clinical findings

**Fig. 2:**
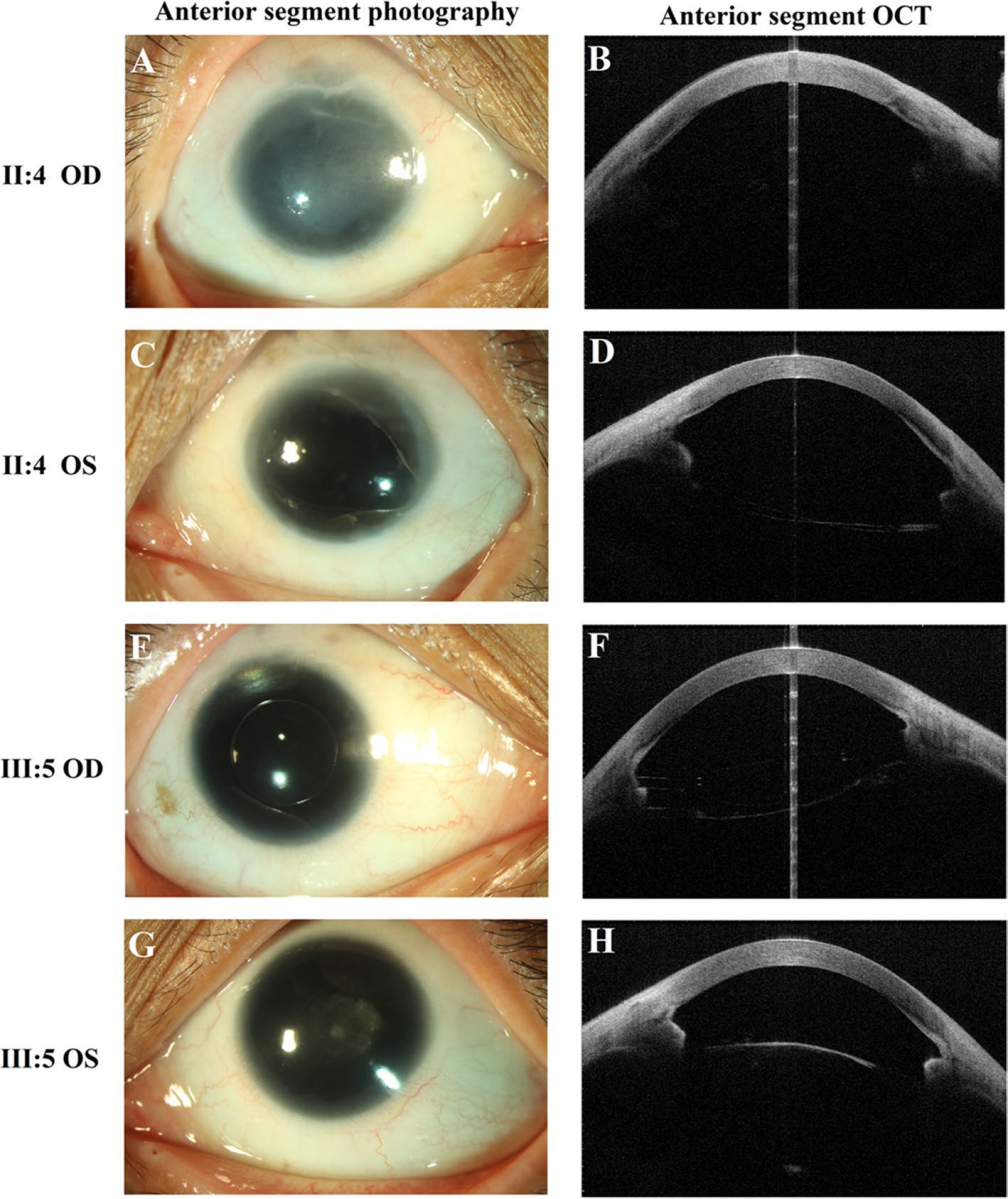
Representative photos of the patients in the family with aniridia. **A**, **C** Anterior segment photography of the proband (II:4) displayed complete aniridia and aphasias in both eyes and corneal leukoplakia in the right eye. **B**, **D** Anterior segment OCT of the proband (II:4) also exhibited total iris absence in both eyes. **E**, **G** Anterior segment photography of the patient III:5 displayed complete aniridia in both eyes, intraocular lens in her right eye and cataract in her left eye. **F**, **H** Anterior segment OCT of the patient III:5 also exhibited total absence of the iris in both eyes

The family investigated in this study shows an autosomal dominant mode of inheritance and is shown in Fig. 1. As illustrated in Table 1, seven affected patients (I:2, II:2, II:4, II:6, III:3, III:5, IV:2) presented with severe visual impairment and glare in both eyes since their early childhood. They received ophthalmic examination and showed similar clinical symptoms, including low visual acuity, aniridia, significant photophobia, nystagmus, cataract (or aphasias, intraocular lens). Furthermore, five patients (I:2, II:2, II:4, III:3, III:5) presented with keratopathy and two patients (II:4, III:3) were found to have binocular glaucoma. Representative photos from anterior segment photography, and anterior segment optical coherence tomography (OCT) of the patients with aniridia are shown in Fig. 2. Some non-ocular symptoms, such as intellectual disability, kidney disease, neurological deficits were not found in the patients. All of the other family members did not have an aniridia phenotype or other major eye diseases.

**Table 1:** Clinical characteristics of the seven patients in this Chinese Han family

Patients	Age, y	Gender	Eye	BCVA	IOP, mmHg	Keratopathy	Aniridia	Nystagmus	Crystalline lens	Glaucoma
I:2	96	F	OD	LP	13	**+**	**+**	**+**	Cataract, Dislocation	**–**
			OS	LP	15	**+**	**+**	**+**	Cataract, Dislocation	**–**
II:2	68	M	OD	20/200	15	**+**	**+**	**+**	Absence^a^	**–**
			OS	HM	17	**+**	**+**	**+**	Absence	**–**
II:4	64	F	OD	LP	16	**+**	**+**	**+**	Absence	**+**
			OS	20/200	14	**–**	**+**	**+**	Absence	**+**
II:6	61	F	OD	20/160	13	**–**	**+**	**+**	Absence	**–**
			OS	20/120	15	**–**	**+**	**+**	Absence	**–**
III:3	37	M	OD	HM	22	**+**	**+**	**+**	IOL	**+**
			OS	HM	23	**+**	**+**	**+**	IOL	**+**
III:5	34	F	OD	20/120	15	**+**	**+**	**+**	IOL	**–**
			OS	20/200	16	**–**	**+**	**+**	Cataract	**–**
IV:2	12	F	OD	20/80	13	**–**	**+**	**+**	Cataract	**–**
			OS	20/100	11	**–**	**+**	**+**	Cataract	**–**

*M* male, *F* female, *OD* the right eye, *OS* the left eye *BCVA* best corrected visual acuity; *LP* light perception, *HM* hand movement, *IOP* intraocular pressure, *IOL* Intraocular lens. All of the other family members have complete iris, without nystagmus or other major eye diseases, and thus are not listed in the table

^a^Absence means the patients had history of phacoemulsification

### Genome sequencing

**Fig. 3:**
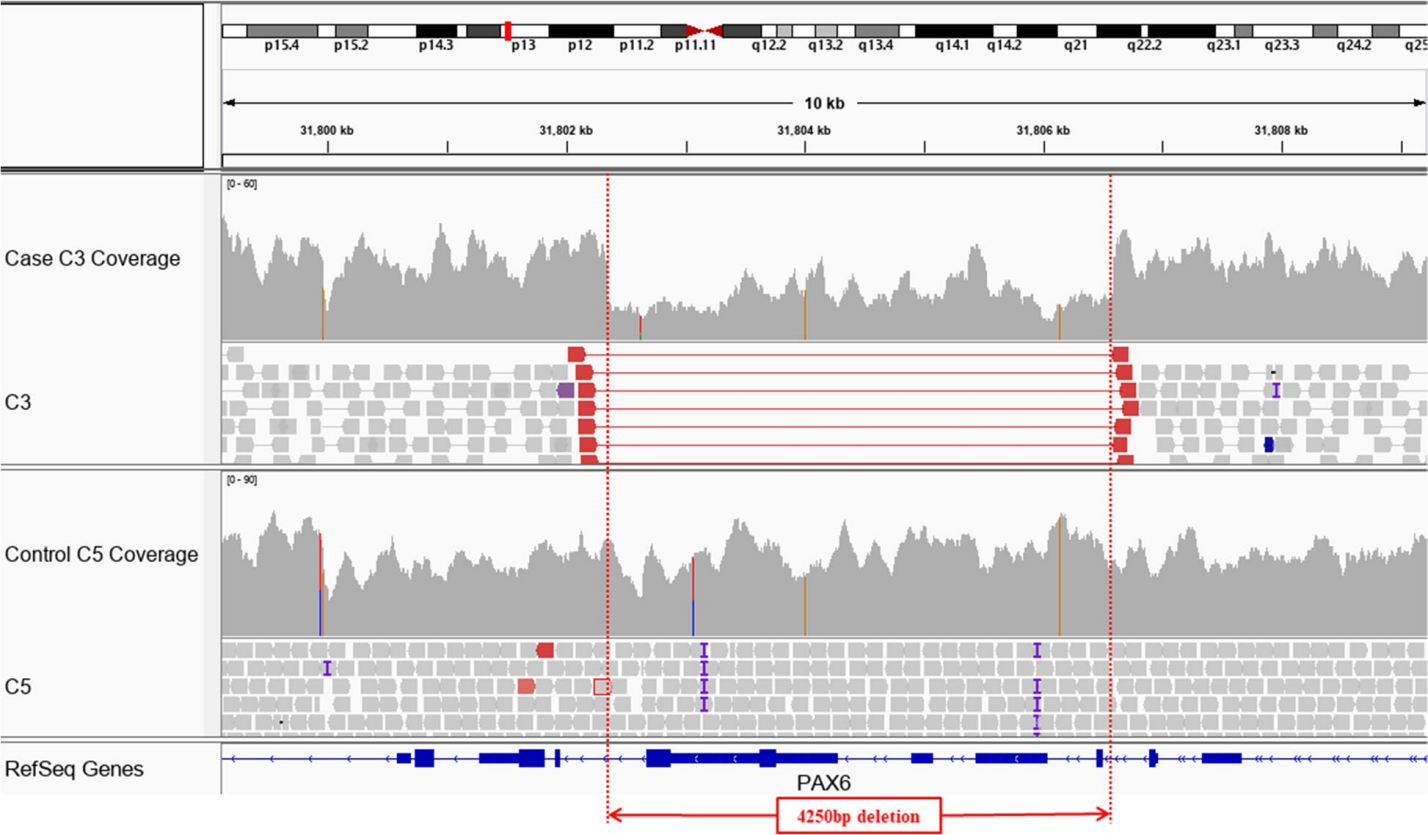
Comparison of high throughput sequencing between affected (and unaffected members by Integrative Genomics Viewer

Filtered variants identified via whole genome sequencing in the affected members were compared with those present in the six healthy individuals. Annotations and filtering of single nucleotide variants (SNVs), indels, structural variants (SVs) and copy number variants (CNVs) were performed shown in Supplementary 1. All the PAX6 variants were provided as Supplementary 2 and no other rare SNV/Indel or SV/Indel was found that is likely to be involved in disease. A ~ 4.25 kb deletion region in *PAX6* gene was detected in affected members that spanned exons 3–4 (NM_000280.5), likely causing abnormal gene translation and/or nonsense mediated decay. This variant is absent from the Database of Genomic variants [21]. *PAX6* variants have previously been shown to be implicated in aniridia [22–24], and as this variant co-segregated with the phenotype, it was considered as causative for disease in the patients. Figure 3 shows comparison of high throughput sequencing between affected and unaffected members by Integrative Genomics Viewer (IGV).

### Validation of a large deletion in PAX6

**Fig. 4:**
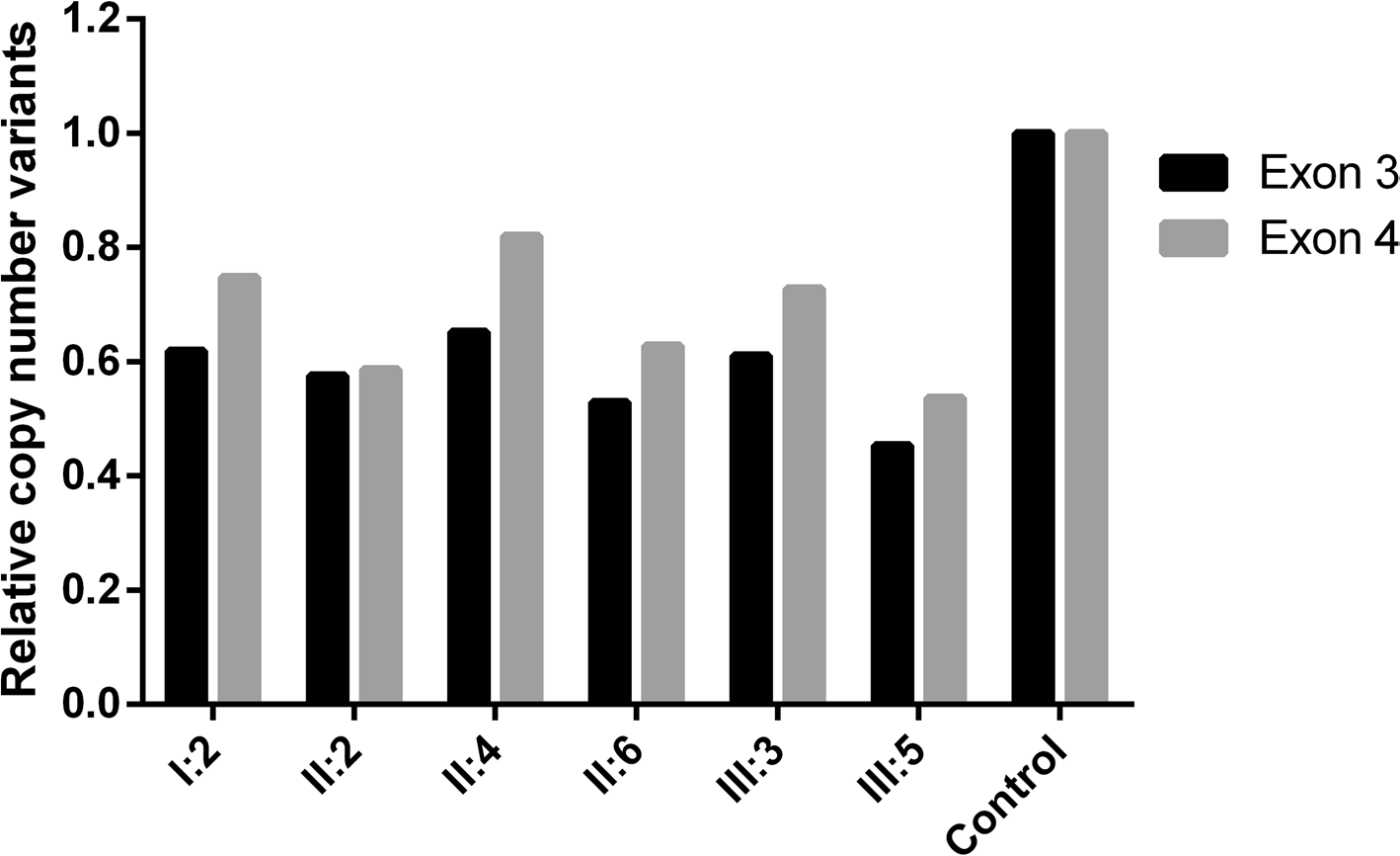
Quantities of exons 3 and 4 by RT-PCR

The remaining DNA of the family was verified by real-time PCR and the results confirmed skipping of exons 3 and 4 (Fig. 4).

**Fig. 5:**
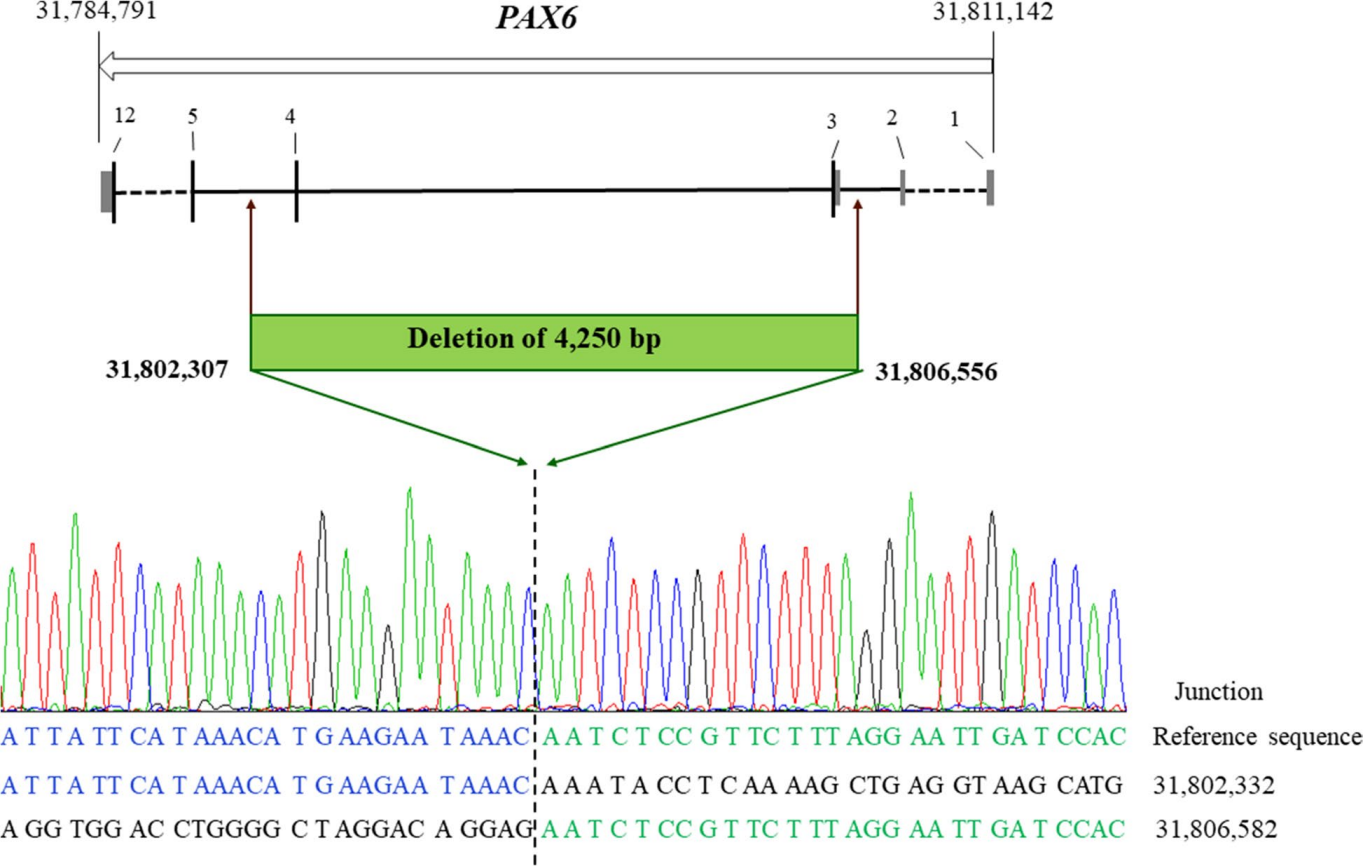
Sequence chromatograms showing the PAX6 deletion mutation identified in this study. The numbers (from 1 to 12) represent exons; black boxes represent coding region and grey boxes represent UTR; Solid lines exons represent introns; intermittent lines represent introns with unequally proportional length

To determine the exact breakpoints of the *PAX6* deletion, we performed PCR and Sanger sequencing using primers flanking the deletion in the patients and unaffected individuals. We verified the presence of a novel 4250 bp heterozygous deletion within the *PAX6* gene, NC_000011.10:g.31802307_31806556del, was identified in all the affected family members (Fig. 5), but not in any of the unaffected members and in the 300 unrelated controls from the same ethnic background. The two breakpoints are located at Intron 4 and the 5′ Untranslated Region (UTR) respectively. The variant was classified as pathogenic based on the guidelines from the American College of Medical Genetics [25, 26].

## Discussion

Congenital aniridia, with or without cataract and nystagmus, is a kind of clinically ocular malformation inherited in an autosomal dominant mode of inheritance with variable expression. *PAX6*, which is located on chromosome 11p13, plays an important role in eye development process by regulating the tissue specific expression of various molecules, structural proteins and related hormones [17]. Most of aniridia cases occur due to a genetic defect of *PAX6* no matter what in sporadic and familial forms. A lot of studies [23, 27–31] have reported that variants in the *PAX6* gene can lead to the clinical symptom of aniridia. Furthermore, *FOXC1* and *PITX2* variants were also associated to aniridia-like phenotypes [32]. The present study identified a novel deletion variant in *PAX6* gene in this Chinese Han family. This finding expands the spectrum of the *PAX6* variants resulting in congenital aniridia.

According to the Leiden Open Variation Database (LOVD, https://www.lovd.nl/) *PAX6* gene database, nearly 90% of disease-causing variants lead to the aniridia phenotype, while the remaining 10% causes follicular dysplasia, Peters Syndrome and small eyeballs [33]. Among these aniridia patients, the clinical manifestations are diverse and aniridia can accompanied with other ocular abnormalities. In this family, corneal leukoplakia or nebula could be found in eight eyes of all fourteen affected eyes (57.1%). The condition of the cornea of patients with aniridia also needs regular examination. Corneal opacification and pannus began peripherally and spreads centrally early in life, which could lead to total opacification of the cornea called aniridia-associated keratopathy (AAK) [34, 35]. Abnormalities of the lens were found in all fourteen affected eyes (100%), manifested cataract and dislocation in this family. Reports of the incidence of glaucoma in cases of aniridia is widely variable, from 6 to 75% [36, 37], and 28.6% in our study. In the early stage, the angle appears open in most cases of aniridia and glaucoma is not present. However, tissue strands containing blood vessels form connections between the iris stroma and the angle wall as time goes on. Once this abnormal iris tissue migrates forward, it might obscure the posterior trabecular meshwork and scleral spur, obstructing the angle and blocking aqueous outflow [38].

The *PAX6* gene spans for approximately 22 kb on chromosome 11p13, contains 14 exons, and encodes two major protein isoforms with either 422 (canonical isoform) or 436 (5a isoform) amino acids [39, 40]. *PAX6* is regulated by multiple enhancers located up to hundreds of kilobases from this locus. Variants in this gene or in the enhancer regions can cause ocular phenotypes and the activity of this protein is essential for the development of neural tissues, particularly the eye [8, 30]. In all affected individuals, the 4.25 kb heterozygous deletion encompasses exons 3 and 4, where the transcription starting site (TSS) is located, which can totally destroy translation initiation or initiation from cryptic sites. Some deletions located downstream of *PAX6* without affecting the coding region are also known to cause aniridia [30, 41], likely affecting downstream regulatory regions.

## Conclusion

In conclusion, a novel 4.25 kb deletion in the *PAX6* gene was found in this Han Chinese family with congenital aniridia combined with cataract and nystagmus. This result expands the mutation spectrum and provides new genetic defects of *PAX6* gene. With the development of genetic analysis, more detailed attention should be required in the clinical consequence of diverse *PAX6* variants.

## Supplementary Information

**Additional file 1.**

**Additional file 2.**

## Data Availability

The datasets generated during the current study are available in the National Genomics Data Center (NGDC) repository, the accession number is HRA000707 and the persistent web link is https://bigd.big.ac.cn/gsa-human/s/dGFiEmrB.

## Abbreviations

PAX6: Paired box gene-6
BCVA: Best corrected visual acuity
IOP: Intraocular pressure
LP: Light perception
HM: Hand movement
IOL: Intraocular lens
OCT: Optical coherence tomography
WGS: Whole genome sequencing
PCR: Polymerase chain reaction
SNV: Single nucleotide variant
SV: Structural variant
CNV: Copy number variant

## References

[1] Nelson LB, Spaeth GL, Nowinski TS, Margo CE, Jackson L (1984). Aniridia. A review. Surv Ophthalmol.

[2] Pozdeyeva NA, Pashtayev NP, Lukin VP, Batkov YN (2005). Artificial iris-lens diaphragm in reconstructive surgery for aniridia and aphakia. J Cataract Refract Surg.

[3] Jordan T, Hanson I, Zaletayev D, Hodgson S, Prosser J, Seawright A, Hastie N, van Heyningen V (1992). The human PAX6 gene is mutated in two patients with aniridia. Nat Genet.

[4] Torkashvand A, Mohebbi M, Hashemi H (2018). A novel PAX6 nonsense mutation identified in an Iranian family with various eye anomalies. J Curr Ophthalmol.

[5] Yahalom C, Sharon D, Dalia E, Simhon SB, Shemesh E, Blumenfeld A (2015). Combined occurrence of autosomal dominant Aniridia and autosomal recessive albinism in several members of a family. Ophthalmic Genet.

[6] Jung R, Rauch A, Salomons GS, Verhoeven NM, Jakobs C, Michael Gibson K, Lachmann E, Sass JO, Trautmann U, Zweier C (2006). Clinical, cytogenetic and molecular characterization of a patient with combined succinic semialdehyde dehydrogenase deficiency and incomplete WAGR syndrome with obesity. Mol Genet Metab.

[7] Lim HT, Kim DH, Kim H (2017). PAX6 aniridia syndrome: clinics, genetics, and therapeutics. Curr Opin Ophthalmol.

[8] Glaser T, Walton DS, Maas RL (1992). Genomic structure, evolutionary conservation and aniridia mutations in the human PAX6 gene. Nat Genet.

[9] Hanson IM, Seawright A, Hardman K, Hodgson S, Zaletayev D, Fekete G, van Heyningen V (1993). PAX6 mutations in aniridia. Hum Mol Genet.

[10] Hingorani M, Hanson I, van Heyningen V (2012). Aniridia. Eur J Hum Genet.

[11] Chalepakis G, Stoykova A, Wijnholds J, Tremblay P, Gruss P (1993). Pax: gene regulators in the developing nervous system. J Neurobiol.

[12] Kim J, Lauderdale JD (2006). Analysis of Pax6 expression using a BAC transgene reveals the presence of a paired-less isoform of Pax6 in the eye and olfactory bulb. Dev Biol.

[13] Grønskov K, Olsen JH, Sand A, Pedersen W, Carlsen N, Bak Jylling AM, Lyngbye T, Brøndum-Nielsen K, Rosenberg T (2001). Population-based risk estimates of Wilms tumor in sporadic aniridia. A comprehensive mutation screening procedure of PAX6 identifies 80% of mutations in aniridia. Hum Genet.

[14] Hu P, Meng L, Ma D, Qiao F, Wang Y, Zhou J, Yi L, Xu Z (2015). A novel 11p13 microdeletion encompassing PAX6 in a Chinese Han family with aniridia, ptosis and mental retardation. Mol Cytogenet.

[15] Robinson DO, Howarth RJ, Williamson KA, van Heyningen V, Beal SJ, Crolla JA (2008). Genetic analysis of chromosome 11p13 and the PAX6 gene in a series of 125 cases referred with aniridia. Am J Med Genet Part A.

[16] Fantes J, Redeker B, Breen M, Boyle S, Brown J, Fletcher J, Jones S, Bickmore W, Fukushima Y, Mannens M (1995). Aniridia-associated cytogenetic rearrangements suggest that a position effect may cause the mutant phenotype. Hum Mol Genet.

[17] Kokotas H, Petersen MB (2010). Clinical and molecular aspects of aniridia. Clin Genet.

[18] Chiang C, Layer RM, Faust GG, Lindberg MR, Rose DB, Garrison EP, Marth GT, Quinlan AR, Hall IM (2015). SpeedSeq: ultra-fast personal genome analysis and interpretation. Nat Methods.

[19] McKenna A, Hanna M, Banks E, Sivachenko A, Cibulskis K, Kernytsky A, Garimella K, Altshuler D, Gabriel S, Daly M (2010). The genome analysis toolkit: a MapReduce framework for analyzing next-generation DNA sequencing data. Genome Res.

[20] Wang K, Li M, Hakonarson H (2010). ANNOVAR: functional annotation of genetic variants from high-throughput sequencing data. Nucleic Acids Res.

[21] MacDonald JR, Ziman R, Yuen RK, Feuk L, Scherer SW (2014). The database of genomic variants: a curated collection of structural variation in the human genome. Nucleic Acids Res.

[22] Ma AS, Grigg JR, Ho G, Prokudin I, Farnsworth E, Holman K, Cheng A, Billson FA, Martin F, Fraser C (2016). Sporadic and familial congenital cataracts: mutational Spectrum and new diagnoses using next-generation sequencing. Hum Mutat.

[23] Park SH, Kim MS, Chae H, Kim Y, Kim M (2012). Molecular analysis of the PAX6 gene for congenital aniridia in the Korean population: identification of four novel mutations. Mol Vis.

[24] Peter NM, Leyland M, Mudhar HS, Lowndes J, Owen KR, Stewart H (2013). PAX6 mutation in association with ptosis, cataract, iris hypoplasia, corneal opacification and diabetes: a new variant of familial aniridia?. Clin Exp Ophthalmol.

[25] Richards S, Aziz N, Bale S, Bick D, Das S, Gastier-Foster J, Grody WW, Hegde M, Lyon E, Spector E (2015). Standards and guidelines for the interpretation of sequence variants: a joint consensus recommendation of the American College of Medical Genetics and Genomics and the Association for Molecular Pathology. Genet Med.

[26] Riggs ER, Andersen EF, Cherry AM, Kantarci S, Kearney H, Patel A, Raca G, Ritter DI, South ST, Thorland EC (2020). Technical standards for the interpretation and reporting of constitutional copy-number variants: a joint consensus recommendation of the American College of Medical Genetics and Genomics (ACMG) and the clinical genome resource (ClinGen). Genet Med.

[27] Cai F, Zhu J, Chen W, Ke T, Wang F, Tu X, Zhang Y, Jin R, Wu X (2010). A novel PAX6 mutation in a large Chinese family with aniridia and congenital cataract. Mol Vis.

[28] Chen P, Zang X, Sun D, Wang Y, Wang Y, Zhao X, Zhang M, Xie L (2013). Mutation analysis of paired box 6 gene in inherited aniridia in northern China. Mol Vis.

[29] Lee PC, Lam HH, Ghani SA, Subrayan V, Chua KH (2014). Investigation of a PAX6 gene mutation in a Malaysian family with congenital aniridia. Genet Mol Res.

[30] Liu X, Wu Y, Miao Z, Zhang H, Gong B, Zhu X, Huang L, Shi Y, Hao F, Ma S (2018). A novel deletion downstream of the PAX6 gene identified in a Chinese family with congenital aniridia. Ophthalmic Genet.

[31] Wawrocka A, Sikora A, Kuszel L, Krawczynski MR (2013). 11p13 deletions can be more frequent than the PAX6 gene point mutations in polish patients with aniridia. J Appl Genet.

[32] Moosajee M, Hingorani M, Moore AT, Adam MP, Ardinger HH, Pagon RA, Wallace SE, Bean LJH, Mirzaa G, Amemiya A (1993). PAX6-Related Aniridia. GeneReviews(®)*.* edn.

[33] Lee HJ, Colby KA (2013). A review of the clinical and genetic aspects of aniridia. Semin Ophthalmol.

[34] Lagali N, Wowra B, Fries FN, Latta L, Moslemani K, Utheim TP, Wylegala E, Seitz B, Käsmann-Kellner B (2020). PAX6 mutational status determines Aniridia-associated Keratopathy phenotype. Ophthalmology.

[35] Mayer KL, Nordlund ML, Schwartz GS, Holland EJ (2003). Keratopathy in congenital aniridia. Ocul Surf.

[36] Ivanov I, Shuper A, Shohat M, Snir M, Weitz R (1995). Aniridia: recent achievements in paediatric practice. Eur J Pediatr.

[37] Swanner JC, Walton DS, Chen TC (2004). Prevention of aniridic glaucoma with goniosurgery. Int Ophthalmol Clin.

[38] Grant WM, Walton DS (1974). Progressive changes in the angle in congenital aniridia, with development of glaucoma. Trans Am Ophthalmol Soc.

[39] Yokoi T, Nishina S, Fukami M, Ogata T, Hosono K, Hotta Y, Azuma N (2016). Genotype-phenotype correlation of PAX6 gene mutations in aniridia. Hum Genome Variation.

[40] Walther C, Gruss P (1991). Pax-6, a murine paired box gene, is expressed in the developing CNS. Development (Cambridge, England).

[41] Simioni M, Vieira TP, Sgardioli IC, Freitas EL, Rosenberg C, Maurer-Morelli CV, Lopes-Cendes I, Fett-Conte AC, Gil-da-Silva-Lopes VL (2012). Insertional translocation of 15q25-q26 into 11p13 and duplication at 8p23.1 characterized by high resolution arrays in a boy with congenital malformations and aniridia. Am J Med Genet Part A.

