# Supplementary material for: A novel 4.25 kb heterozygous deletion in PAX6 in a Chinese Han family with congenital aniridia combined with cataract and nystagmus
Source: BMC Ophthalmol. 2021 Oct 5;21:353. doi: 10.1186/s12886-021-02120-0 (PMC8491394; doi:10.1186/s12886-021-02120-0)

**A Novel 4.25kb Heterozygous Deletion in *PAX6* in A Chinese Han Family with Congenital Aniridia Combined with Cataract and Nystagmus**

Tianwei Qian^1-7,*^, Chong Chen^1-5,*^, Caihua Li^8^, Qiaoyun Gong^1-5^, Kun Liu^1-5^, Gao Wang^6^, Isabelle Schrauwen^6^, Xun Xu^1-5^

^1^ Department of Ophthalmology, Shanghai General Hospital, Shanghai Jiao Tong University, Shanghai, China

^2^ National Clinical Research Center for Eye Diseases, Shanghai, China

^3^ Shanghai Key Laboratory of Ocular Fundus Diseases, Shanghai, China

^4^ Shanghai Engineering Center for Visual Science and Photomedicine, Shanghai, China

^5^ Shanghai Engineering Center for Precise Diagnosis and Treatment of Eye Disease, Shanghai, China

^6^ Department of Neurology, Columbia University Medical Center, New York, USA

^7^ Singapore Eye Research Institute, Singapore National Eye Centre, Singapore, Singapore

^8^ Genesky Biotechnologies Inc, Shanghai, China

**Supplementary 1**

Filtering strategy of variants identified via whole genome sequencing. (A) single nucleotide variants/indels; (B) structural variants; (C) copy number variants.


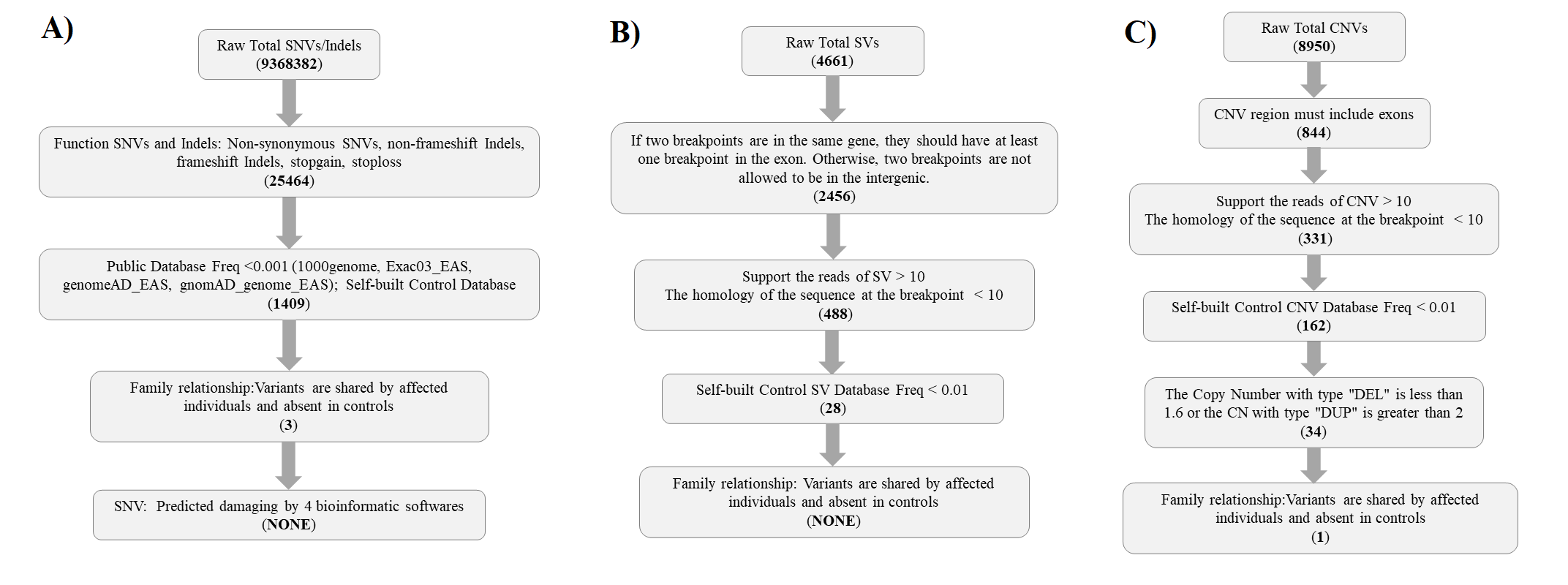

Supplement: Supplementary file 1 — Additional file 1. [file 12886_2021_2120_MOESM1_ESM.docx]
